# Memory and selective learning in children with spina bifida-myelomeningocele and shunted hydrocephalus: A preliminary study

**DOI:** 10.1186/1743-8454-2-10

**Published:** 2005-11-17

**Authors:** Behroze Vachha, Richard C Adams

**Affiliations:** 1 Pediatric Developmental Disabilities, Texas Scottish Rite Hospital for Children, Dallas, TX, 75219, USA; 2 Department of Pediatrics, University of Texas Southwestern Medical Center, Dallas, TX, 75235, USA

## Abstract

**Background:**

Selective learning is the ability to select items of relevance from among less important items. Limited evidence exists regarding the efficiency with which children with spina bifida-myelomeningocele and shunted hydrocephalus (SB/SH) are able to learn information. This report describes initial data related to components of learning and metacognitive skills in children with SB/SH.

**Methods:**

Twenty six children with SB/SH and 26 controls (age: 7 – 16 y) with average intelligence, and monolingual English-speaking backgrounds participated in the study. Exclusion criteria for the SB/SH group were: prior history of shunt infection, history of seizure or shunt malfunction within the previous three months, prior diagnoses of attention disorders and/or clinical depression. Children were presented lists of words with equal exemplars each of two distinct semantic categories (e.g. fruits, animals), and told to make as high a score as possible by learning the words. The value of the words was designated by category membership (e.g. animals = low value; fruits = high value). The total number of words learned across three learning trials was used to determine memory span. Selective learning efficiency (SLE) was computed as the efficiency with which items of greater value were selectively learned across three trials.

**Results:**

Children with SB/SH did worse than controls on memory span (*P *< 0.05). Although SLE was not significantly different between groups, when asked what strategy was used in the selective learning tasks, 65% of the SB/SH children said they tried to remember all words (inefficient strategy). In contrast, 85% of controls said they tried to remember the higher value words – the more efficient strategy.

**Conclusion:**

Success in school is often dependent on the ability to recall important facts selectively and ignore less important information. Children with SB/SH in our study had a poor memory span and were unable to monitor and report an efficient and workable metacognitive strategy required to remember a list of words. Preliminary findings may begin to explain our previous clinical and research findings wherein children with SB/SH often focus on extraneous details, but demonstrate difficulty remembering the main gist of a story/event.

## Background

The potential barriers capable of impacting daily learning, recall, and functional application of prior knowledge (in rehearsed or novel situations) among children with spina bifida-myelomeningocele and shunted hydrocephalus (SB/SH) are multifaceted and tightly inter-related. Neurological insults include ventriculomegaly of varying degrees, callosal formation of varying completion, Chiari II malformation with varying complexity, and others [1]. By their nature, these neurological differences occur along a continuum rather than a simple, discrete, all-or-none classification.

Variations in cognitive functions impacting learning among children with SB/SH are similarly complex. The overwhelming majority of such youngsters have average intelligence based on standardized tests [2]. Prior studies have described potential barriers to learning in this population based on differences in language abilities and in temperament characteristics [3,4]. Given the underlying developmental differences in brain structure, other cognitive skills such as memory, as well as metacognitive skills, might be expected to contribute to the learning difficulties demonstrated by these children.

We report here a preliminary study of memory and metacognitive skills, specifically 'selective learning' abilities, in children with SB/SH and their impact on learning. Selective learning (SL) has been defined as the ability to select and learn particular items of higher value from a broader array of available information [5]. This process has been described in typically developing children (6 to 16 years) and among adults and children with post-natal traumatic brain injury (TBI) of various etiologies [5-8]. Among individuals with TBI, a better understanding of how the child subsequently selects, focuses on, and learns salient facts may be central to success both within the classroom and within home / community settings. In each of these environments, everyday circumstances require quick and appropriate responses.

For children with SB/SH, brain abnormalities begin in the first month of gestation and are sustained beyond birth [1,9-11]. These developmental disabilities are fundamentally different from those of children with previously typical development who subsequently sustain brain injury. Nevertheless, their ability to learn salient information selectively is at least as critical for successful performance both in academic and life skills. Since memory capacity limits the ability to recall all information encountered, successful classroom learning is dependent, in part, on the child's ability to selectively learn and recall the important facts in comparison to less important information [12]. Studying selective learning and metacognitive learning strategies employed by children with SB/SH should provide professionals (rehabilitation and school teachers, alike) important insights into an individual child's specific strengths and weaknesses so that strategies/methods of teaching can be optimized.

The primary aim of this study was to investigate the ability of children with SB/SH to learn selectively items based on differential value in comparison to typically developing peers. Additionally, in order to determine if there were dissociations within the same task between the total words recalled without regard to value (memory span) and the more complex processes involved in selective learning efficiency, we investigated differences in the memory span of children with SB/SH and their controls. Finally, we computed and compared the number of children in each group that reported the most efficient strategy that might help them learn and remember the items in order to be most successful in a learning task.

## Methods

### Participants

Twenty-six children with SB/SH between the ages of 7–16 years (*M = *12.3y, *SD *= 2.7) were recruited from the Spina Bifida Program at Texas Scottish Rite Hospital for Children (TSRHC), Dallas, Texas. Twenty-six typically developing children from the same geographic region, aged 7–16 years (*M *= 11.2y, *SD *= 2.6) served as controls. Research reported in the manuscript was performed with the approval of the ethics committee of the Institutional Review Board at the Texas Scottish Rite Hospital for Children and University of Texas Southwestern Medical Center, Dallas and was in compliance with the Helsinki declaration (Permission Number: 0603-421).

To minimize confounding variables, strict inclusion and exclusion criteria were created and followed. Specifically, inclusion criteria for the SB/SH group included: 1) Diagnosis of myelomeningocele with Chiari II; 2) Hydrocephalus diagnosed, and VP shunt placed within the first year of life. Inclusion criteria for both the SB/SH and control groups included: 1) English as primary language; 2) No history of seizures within the past three months. 3) Average intelligence (Full Scale IQ greater than 80 on the Wechsler Intelligence Scale for Children – Fourth Edition). Exclusion criteria for the SB/SH group were: 1) History of shunt infection; 2) Shunt malfunction with resultant symptoms within the previous three months. Exclusionary criteria for both groups included: 1) Uncorrected sensory (auditory or visual) acuity deficits or motor disabilities that would preclude a pointing response; 2) Prior diagnosis of mental retardation; 3) Prior diagnosis of attention deficit disorder.

### Procedure

Children were presented lists of 14 spoken words comprising seven exemplars each of two distinct semantic categories (e.g. fruits and animals). Category exemplars for both categories comprised concrete, low age of acquisition (AoA) words drawn from the University of Western Australia Psycholinguistics Database [13], from a set of 297 object names normed by age of acquisition reported by Morrison et al. [14] and from samples of learning materials prescribed for kindergarteners in the Texas public education system [15]. AoA rating for words was set for 22 – 60 months of age. To further ensure that children in this study would have been exposed to these exemplars by age 5 years in the Texas public education system, selected words were rated for age of acquisition by a panel of three educators in the Texas public education system, each with at least six years of kindergarten to first grade teaching experience. If a discrepancy in concreteness or AoA rating for a word could not be resolved between the three educators, that word was discarded (for e.g. bear, bat having two meanings were removed from the original listing), yielding the final set of words deemed to be acquired by a majority of children by 5 years of age. Categories were counterbalanced such that if an exemplar in one category had a particular age of acquisition, then an exemplar with a similar age of acquisition was selected for the other category.

Each child was individually tested, and the list was spoken by a single examiner at the rate of one word per second. There was one practice trial and a total of three consecutive experimental trials presented in the same session.

Each child was told to make as high a score as possible by remembering the words. The value of the words was designated by category membership (e.g. name of any fruit = 10 points; name of any animal = 1 point). For each list, the value assigned to the categories was told to the child both prior to list presentation and after list presentation, immediately before recall, in order to eliminate memory for category-value designations as a confounding variable. The words were mixed in each list so that exemplars for the two categories occurred in random order within the list.

To ensure that the children were equally familiar with exemplars in both categories, each child was given a post-test screening task in which he/she was asked to: 1) name the category exemplars used in the experiment (shown to them as colored pictures); and 2) sort the category exemplars used in the experiment into the appropriate categories.

Colored pictures representing exemplars in each category were mixed randomly and presented in the same sequence to each child. One picture was presented at a time in the center of a computer screen. Participants were instructed to name each picture as quickly but as accurately as possible. Each picture remained on the screen until the child named it. As soon as the participant responded, the picture disappeared and a blank screen was presented for 1500 msec, followed by the next picture. Correct and incorrect responses were recorded manually on a record form. Response time was recorded in msec from onset of picture presentation to onset of response. The total number of correct responses for each category was computed and response times were calculated for correct responses only. If a child self-corrected an incorrect response, it was counted towards the total number of correct responses made by the child, but was excluded from response time calculation.

Given the low age of acquisition of the tests, children in both groups were able to name all category exemplars used in the experiment with 100% accuracy. SB/SH and control groups did not differ significantly in mean response times for any category (*P *> 0.05). Within group analysis showed children with SB/SH did not demonstrated significant differences in mean response time for naming exemplars in either category (*P *> 0.05). Children in the control group also did not demonstrated significant differences in mean response time for naming exemplars in either category (*P *> 0.05).

Next, the same sequence was presented, but this time the child was asked to provide the category for the exemplar. Correct and incorrect responses were recorded manually. The total number of correct responses for each category was computed and response times were calculated as stated above. Children in both groups were able to sort the category exemplars used in the experiment into the appropriate categories with 100% accuracy. Between and within group analysis demonstrated no significant differences in mean response time for sorting category exemplars for the two groups (*P *> 0.05).

### Scoring

There were three main measures of interest. The first was the degree of selective learning efficiency demonstrated by the child learning preferentially the words of higher value (e.g. fruits) in comparison to words of lower value (e.g. animals). To ensure that the measure be independent of the total number of words recalled (i.e. the child's memory span), we calculated the selective learning efficiency of each trial for each subject using the following formula as given by Hanten and colleagues [5] : Selective learning efficiency (SLE) = (actual score - chance score)/(ideal score - chance score), where the *actual *score = the sum of the values of the items recalled; the *chance *score = the score that is predicted in the absence of selectivity given the number of items recalled (in other words, if half the words recalled were high value and half were low value); *ideal *score = maximum score that could be achieved given the number of items recalled. The range of the SLE index extends from -1 to 1 with a score of 0 indicating chance performance.

The second measure of interest was the total number of words recalled, which reflects the memory span of the child. This measure was the average number of words recalled across trials without regard to the word value and included the total number of high and low value words averaged across three trials.

The third measure of interest examined whether children reported the most efficient strategy needed to learn words in order to make the highest score possible on the selective learning task. Children in both groups were asked what would be the most efficient strategy required to remember the words in order to make the highest score on the learning task. Responses were recorded and coded 'efficient' if the response was "Try to remember high value word (e.g. fruits) more than low value word (e.g. animals)." or similar variant. Responses were considered 'inefficient' if the response was "I tried to remember all words while you said them" or similar variant. The percentage of children reporting the efficient versus inefficient strategies for each group was calculated.

### Results

Preliminary analyses showed no significant group differences in age (*t *_[50] _= -1.47, *P* = 0.148), mean parental education, as proxy for socioeconomic status, (*t *_ [50] _= 0.64, *P* = 0.526); gender (χ^2^_[1] _= 0.080, *P *= 0.778); or ethnicity (χ^2^_[2] _= 3.476, *P* = 0.176). No significant correlations were noted between mean parental education and mean SLE (*P* = 0.93) or mean memory span (*P* = 0.58). No significant differences were noted in performance by males versus females on measures of mean SLE (*t *_ [50] _= 0.55, *P* = 0.58) or mean memory span (*t *_ [50] _= 0.29, *P* = 0.77).

### SLE

The mean SLE, not adjusted for age, for the SB/SH and control groups is provided in Table 1. Although the children with SB/SH appeared to have lower mean SLE averaged across the three trials than did typically developing children, the group differences failed to reach significance (*F*_ [1,50] _= 0.98, *P* = 0.327). Pearson correlations indicated that age-at-test of the child was positively related to mean SLE (*r *= 0.36, *P* = 0.01) such that as the child's age increased, the SLE increased. Therefore, mean SLE averaged across the three learning trials was analyzed using analysis of covariance with group (SB/SH or control) as the independent variable and age-at-test as covariate, at a *P*value of 0.05. No significant differences were noted in the SLE/age relationship as a function of group (age*group interaction *F *_ [1,48] _= 1.73, *P *= 0.20). After adjusting for age, the differences between groups remained non significant (*F*_ [1,49] _= 2.81, *P *= 0.10). There was a significant effect of age (*F*_ [1,49] _= 9.56, *P *= 0.003).

**Table 1:** Age, SLE index and memory span for children with SB/SH and controls. Data are means (SD).

**Group**	**Age (years)**	**SLE index**	**Memory span**
SB/SH	12.3 (2.7)	0.29 (0.34)	6.9 (1.9)
Control	11.2 (2.6)	0.39 (0.32)	8.9 (2.0)

A repeated measures analysis of covariance was used to compare the two groups (SB/SH or control), and evaluate consistency across the three trials for SLE, using age as covariate. The repeated measures analysis of covariance using Wilk's Lambda found no significant differences across the three trials (*F *_ [2,48] _= 1.58, *P *= 0.22), a non significant interaction of trial*age (*F *_ [2,48] _= 1.14, *P *= 0.33), and a non significant interaction of trial*group (*F *_ [2,48] _= 0.30, *P *= 0.74).

### Memory Span

The mean memory span, not adjusted for age, for the SB/SH and control groups is provided in Table 1. A significant effect of group indicated children with SB/SH had a significantly lower mean memory span averaged across the three trials (*F*_ [1,50] _= 14.11, *P* < 0.001). Group means, not corrected for age are reported in Table 1. Pearson correlations indicated that age-at-test of the child was positively related to mean memory span (r = 0.40, *P* = 0.003) such that as the child's age increased, the memory span also increased. Therefore mean memory span averaged across the three learning trials was analyzed using analysis of covariance with group (SB/SH or control) as the independent variable and age-at-test as covariate, at a *P *value of 0.05. No significant differences were noted in the memory span/age relationship as a function of group (age*group interaction *F*_ [1,48] _= 0.05, *P* = 0.82). After adjusting for age, there remained a significant difference between the two groups (*F*_ [1,49] _= 29.76, *P* << 0.001), with controls having a higher mean than SB/SH children.

The repeated measures analysis of covariance with age as covariate and using Wilk's Lambda found no significant differences across the three trials (*F *_ [2,48] _= 0.29, *P *= 0.75), a non significant interaction of trial*age (*F *_ [2,48] _= 0.29, *P *= 0.75), and a non significant interaction of trial*group (*F *_ [2,48] _= 1.77, *P *= 0.18).

### Metacognitive Strategy

**Figure 1:**
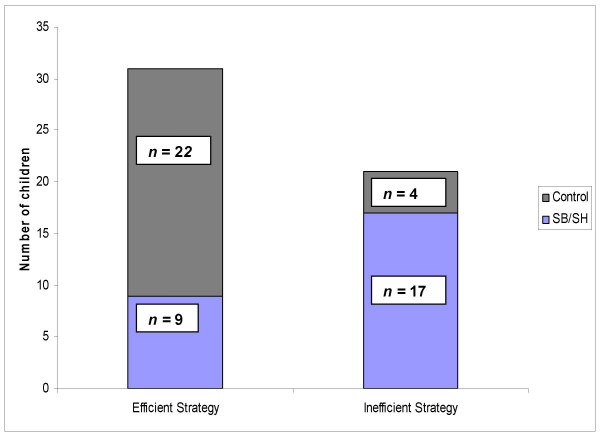
Efficient/inefficient strategy reported by children with SB/SH and controls.

The majority of children with SB/SH were unable to monitor and report the most efficient strategy they should use in order to learn the words in a fashion that would support their making the highest possible score on the task. The majority of them (65%) reported that they tried to remember all the words equally as they perceived that to be the strategy that would most likely yield a high score (inefficient strategy, Fig. 1). In contrast, most children in the control group (85%) said they tried to remember the *fruits *more than the *animals *because fruits were worth much more than the animals (efficient strategy, Fig. 1). Statistical analysis (two-tailed Fisher Exact Test) confirmed that children with SB/SH were unable to monitor and report the learning strategy most likely to yield the highest score on the selective learning task (two-tailed *P *< 0.001).

## Discussion

A finding of this preliminary study was that although children with SB/SH appeared to have lower SLE averaged across the three trials than did typically developing children (Table 1), group differences failed to reach significance. This result may be attributed to the relatively small sample size used in the study. However, a more likely explanation may be related to the poor memory span demonstrated by children with SB/SH.

Consistent with previous studies of learning and memory in children with SB/SH [16,17] children with SB/SH in this study demonstrated significantly impaired memory span. In other words, children with SB/SH remembered fewer words overall irrespective of the value (higher or lower) of the words, suggesting the lack of significance on SLE tasks may be related to a pervasive memory impairment in this group.

Our findings are directly opposed to those seen in recent studies in children with TBI which showed impaired SLE but preserved memory span [5,6]. Budson and Price [18] recently reviewed different memory systems and their dependence on different neuroanatomical structures and variations. They described working memory (a component of selective learning) as "the ability to temporarily maintain and manipulate information that one needs to keep in mind" and dependent on "a network of activity that includes subcortical structures as well as frontal and parietal cortical regions" [18]. Just as injury to the orbitofrontal circuits secondary to trauma may play a role in differentially affecting more than one memory system in TBI [5,6], developmental variations unique to SB/SH might be expected to impact multiple, but potentially different components of the central nervous system than those involved in children with TBI. These individually and collectively, may impact learning and subsequent academic performance.

In addition to poor memory span performance, the results of this study also suggested that children with SB/SH were impaired relative to typically developing children in their ability to report the most efficient strategy in learning items of differential value in order to attain the highest possible score on a learning task. Learning and memory skills are inextricably linked and are critical components for optimal academic performance. Overt memory deficits are likely to be noted in neuropsychological evaluations, and when these children encounter professionals/parents within medical, school or home settings. Less dramatic, but equally important variations in the child's perception and recognition of useful learning strategies can also contribute to functional impairments within these environments. For instance, school-based texts have salient elements interspersed with less relevant information included in the service of making the text more interesting to learners. Success in school is potentially dependent on the ability to selectively learn and recall important facts and ignore less important information [5]. Educational teaching strategies suggest that an effective intervention for fact learning is to point out the salient components to be learned and provide incentives for learning [19]. The majority of children with SB/SH in our study were unable to reason out and report the incentive inherent in the selective learning task utilized in this study (i.e. more points for high value words than low value words). These preliminary findings are consistent with our previous clinical and research findings wherein children with SB/SH focus on many extraneous details, but are unable to remember the salient points of a story/event [20,21]. The results suggest a difficulty with metacognitive skills or the ability to think about appropriate learning strategies. Automatic strategies such as trying to remember as much information as possible regardless of value, taken together with the fact that these children already have an impaired memory span may account for the less than optimal academic performance described in these youngsters and warrants further investigation.

To ensure familiarity with exemplars in each category, children were presented with lists of words of low age of acquisition and concreteness. Post-test screening also revealed children in both groups were able to rapidly name and sort test items into the correct category with 100% accuracy. The category designated as high value was not, however, counterbalanced by subject in this study. While unlikely, it is possible that an individual child's preference of animals over fruits (or vice versa) could conceivably have resulted in a tendency bearing on his selective learning performance. This issue warrants consideration in future studies. Future studies that determine differences in selective learning performance based on whether words were presented auditorily or visually or both will help elucidate the types of memory and cognitive processes affected in children with SB/SH.

In summary, mood, anxiety, language and its various subsystem components, cognition (attention, abstract reasoning), temperament, family and environmental variables, and underlying autonomic instability have all been described among children with SB/SH [3,4,20,22]. Each of the above contributes to or detracts from the process of 'learning' whether in the classroom, or in the community. The concept of a 'spina bifida-hydrocephalus learning spectrum' might be more functional to parents, students, and educators than attempts to identify a single profile. To this spectrum, the various subsystems of memory and metacognition must be added. Further studies in each of these component arenas are likely to underscore the complexity of the child with SB/SH, but should allow the informed professional to bring useful information to assist the child in achieving his or her maximum learning potential.

## List of abbreviations

SB/SH, shunted hydrocephalus and spina bifida-myelomeningocele; SLE, selective learning efficiency; AoA, age of acquisition

## Competing interests

The author(s) declare that they have no competing interests.

## Authors' contributions

Both authors contributed equally to this work. All authors read and approved the final manuscript.
